# Macular Variability in Children and Adolescents with Metabolic Syndrome: A Cross-sectional Study Examining the Associations with Anthropometric Measurements, Metabolic Parameters and Inflammatory Markers

**DOI:** 10.4274/jcrpe.galenos.2019.2019.0082

**Published:** 2020-03-19

**Authors:** Hakan Öztürk, Bediz Özen, Gönül Çatlı, Bumin N. Dündar

**Affiliations:** 1 University of Health Sciences Turkey, İzmir Tepecik Training and Research Hospital, Clinic of Ophthalmology, İzmir, Turkey; 2 İzmir Katip Çelebi University Faculty of Medicine, Department of Pediatric Endocrinology, İzmir, Turkey

**Keywords:** Macular retinal thickness, macular retinal volume, metabolic syndrome, optical coherence tomography, pediatric obesity

## Abstract

**Objective:**

Macular damage may be observed in obesity and metabolic syndrome (MetS), a condition which leads to chronic subclinical inflammation and affects most organ systems. To investigate the association between macular variability and anthropometric measurements, metabolic parameters, and inflammatory markers in children and adolescents with MetS.

**Methods:**

Two hundred and twenty eyes of 62 obese and 48 healthy children and adolescents were examined. Bilateral macular retinal thickness (MRT) and macular retinal volume (MRV) were measured in all subjects using optical coherence tomography. Associations between mean MRT and mean MRV and age, auxological measurements including body mass index standard deviation scores (BMI-SDS) and waist circumference-SDS (WC-SDS), metabolic parameters and inflammatory parameters including neutrophil/lymphocyte ratio (NLR), platelet/lymphocyte ratio and systemic immune-inflammatory index (SIII) were investigated.

**Results:**

No statistically significant difference was observed between the groups in terms of age or sex distribution (p>0.05). Mean MRT (r=-0.326, p=0.007) and MRV (r=-0.303, p=0.007) values in the obese group with MetS decreased as homeostasis model assessment-insulin resistance (HOMA-IR) values increased. SIII values were higher in obese groups, but particularly in obese subject with MetS, compared to the control group (p=0.021). The decrease in mean MRT (r=-0.544, p=0.046) and MRV (r=-0.651, p=0.031) in the obese subjects with MetS was negatively correlated with NLR. Mean MRT and MRV decreased in all obese subjects as SIII increased (p<0.05).

**Conclusion:**

This is the first study to show that mean MRT and MRV values decrease as BMI-SDS, WC-SDS and HOMA-IR increase in obese children and adolescents with MetS. NLR and SIII may serve as markers of chronic inflammation in obese children with MetS associated with macular damage.

**What is already known on this topic?**

Obesity and metabolic syndrome (MetS) are capable of causing damage to several organ systems by triggering a chronic subclinical inflammatory process. In the eye this damage includes microangiopathic changes, retinal degeneration, optic nerve function impairment and damage in the choroid and macular regions. Optical coherence tomography may show early macular damage.

**What this study adds?**

This is the first study to show that macular retinal thickness and macular retinal volume values decrease as body mass index-standard deviation scores (SDS) and waist circumference-SDS (WC-SDS) increase in obese children and adolescents with MetS, providing evidence of macular damage. The increases in neutrophil/lymphocyte ratio, the platelet/lymphocyte ratio and the systemic immune-inflammatory index may be markers of chronic inflammation in children with MetS, and are associated with macular damage.

## Introduction

The prevalence of obesity and metabolic syndrome (MetS), usually a complication of obesity, is increasing. MetS is capable of causing damage to several organ systems by triggering a chronic subclinical inflammatory process. In the eye this damage includes microangiopathic changes, retinal degeneration, optic nerve function impairment and damage in the choroid and macular regions (1,2). These changes can be identified in the early stages using optical coherence tomography (OCT). The layers of the eye can be visualized in a painless, rapid, and non-invasive manner with OCT (3). OCT is particularly used to visualize macular pathologies, such as diabetic macular edema and macular degeneration (4,5,6). The macula is the region of the eye which is critical for detailed vision and is of great importance to the sense of sight. A deleterious effect in the macular region can cause progressive or permanent vision impairment. The macula can also be damaged in obesity and MetS because of the accompanying chronic subclinical inflammation (7).

Complete blood count is an inexpensive and easily accessible test. The neutrophil/lymphocyte ratio (NLR), platelet/lymphocyte ratio (PLR) and systemic immune-inflammatory index (SIII), all of which are easily calculated from complete blood count, have been shown to indicate subclinical inflammation in several studies (8,9,10).

To the best of our knowledge, no previous studies have investigated the association between changes in macular retinal thickness (MRT) and macular retinal volume (MRV) with metabolic parameters nor with inflammatory markers in children with MetS. The purpose of this study was to use OCT to investigate variability in MRT and MRV between obese children and adolescents with and without MetS and healthy controls as evidence of macular damage. This study also evaluated associations between changes in macular retinal measurements and anthropometric measurements, metabolic parameters, pubertal stage, and NLR, PLR and SIII.

## Methods

This prospective observational study was undertaken after receipt of Institutional Medical Research Ethical Committee approval (2019/9-3). The study was conducted in accordance with the ethical principles of the Declaration of Helsinki.

Patients aged 10-18 years, with no history of ocular disease or surgery, with no neurological diseases, and with spherical values between -0.75 diopter (D) and +0.75 D were enrolled in the study and control groups. All children and their parents consented to participate.

Children with a history of ocular trauma and dense media opacities, use of systemic corticosteroids, with diabetes mellitus or any systemic disease, or with acute/chronic local/systemic infectious disease and those unsuitable for OCT measurement, were excluded.

One hundred and twenty-four eyes of 62 obese children and adolescents aged between 10.1 and 17.9 years who presented to İzmir Tepecik Training and Research Hospital Pediatric Endocrinology Clinic, Turkey, between February 2016 and February 2019 were included in the study. Controls consisted of the 96 eyes of 48 healthy children and adolescents aged between 10.0 and 17.8 years presenting during the same period. The controls were composed of healthy children who applied to the ophthalmology clinic for routine control and without obesity.

Demographic data for all groups were extracted from medical records. Body measurements, blood pressure values and pubertal stages were assessed by a pediatric endocrinologist. Pubertal stages were classified based on the Tanner system (11). Height was measured to the 0.1 cm nearest centimeter with a rigid stadiometer. All subjects were also weighed unclothed to the nearest 0.1 kg using a calibrated balance scale. Body mass index (BMI) was calculated using the standard formula; weight (kg)/height (m^2^). Standard deviation scores (SDS) for weight, height and BMI were calculated based on reference values established for Turkish children (12). Obesity was diagnosed according to the World Health Organization criteria (13). Blood pressure was measured three times at 10 minute intervals following a rest period. Systolic and/or diastolic blood pressure values exceeding the 95^th^ percentile were regarded as hypertensive (14). In the obese subjects complete blood count, blood glucose, insulin and serum lipids were measured from fasting venous specimens collected on the same day as anthropometric measurements were taken. Specimens were analyzed using Roche Hitachi Modular System (Mannheim, Germany). Insulin resistance (IR) assessed by homeostasis model assessment-IR (HOMA-IR) was calculated with the formula; fasting insulin (µIU/mL) × fasting glucose (mg/dL)/405 (15). MetS was diagnosed based on International Diabetes Federation criteria (16). The syndrome was defined as hypertriglyceridemia (>150 mg/dL), decrease in high density lipoprotein (HDL) (<40 mg/dL), blood pressure elevation (systolic blood pressure ≥130 mmHg, diastolic blood pressure ≥85 mmHg), or glucose metabolism disorder in the presence of abdominal obesity in cases aged 10-16 years. Adult criteria were used for the diagnosis of MetS at the age of 16 years and over. Percentile curves established for Turkish children were used for waist circumference. A waist circumference of the ≥90^th^ percentile was regarded as central obesity (17). The online calculator program developed by Demir et al (18) was used for all auxological measurements and blood pressure evaluation.

Three groups were established – healthy control (group 1), MetS negative (MetS-) obese (group 2), and MetS positive (MetS+) obese (group 3).

All cases underwent extensive ocular examination by the same ophthalmologist. This included best corrected visual acuity, detailed anterior segment examination with slit-lamp biomicroscopy, intraocular pressure using a Goldman applanation tonometer, ocular motility examination and optic nerve and retina examination with a 90 D lens. One percent cyclopentolate hydrochloride (Sikloplejin - Abdi İbrahim İlaç Sanayi, İstanbul, Turkey) eye drops were administered three times at five minute intervals for pupillary dilation. Measurements were recorded using an automated refractor (Topcon KR-1, Topcon, Tokyo, Japan) 30 minutes after the final drop administration. The mean value of the three measurements was recorded.

Retinal thickness and volume in the macular region were measured using a Spectralis OCT device (Heidelberg Spectralis, Heidelberg Engineering, Heidelberg, Germany). All participants were asked to wait in a darkened room before measurement. All scans were carried out on a 20 x 20 degree cube with 49 raster lines at 120-µm intervals. Retinal thickness and volume in the macula were calculated automatically as the distance separating the vitreoretinal interface from the margin representing the junction of the photoreceptor inner and outer segments. The macula was divided automatically into three concentric 1-, 3- and 6-mm rings. Retinal thickness and volume were measured on these three rings in accordance with the Early Treatment Diabetic Retinopathy Study Groups macular map (19). The inner and outer rings were further divided into four quadrants (superior, temporal, inferior, and nasal) by two reticules.

The 1-mm ring was defined as the central circle, the 3-mm ring as the inner segment circle and the 6-mm ring as the outer segment circle. Ocular observation was performed automatically in real time. Measurements were taken from both eyes by two independent masked observers. In order to avoid diurnal fluctuations, all OCT images were taken between 10.00 and 12.00 hours. Total macular thickness and volume including all nine subfields were subjected to analysis.

The MRT and MRV values were subjected to statistical analysis and comparison between the groups. In addition, correlation analysis was performed between mean MRT and MRV values and age, pubertal stages, body measurements, systolic/diastolic blood pressures, fasting insulin, HOMA-IR, lipid values, NLR, PLR, and SIII. SIII, was calculated using the formula (platelet × neutrophil)/lymphocyte.

### Statistical Analyses

Statistical analysis was performed on Statistical Package for Social Sciences (SPSS 20.0; IBM, Chicago, Ill., USA) software. Normality of sample distribution was evaluated using the Kolmogorov-Smirnov test. Mean and standard deviation values were calculated for normally distributed data. Pearson correlation analysis was applied for normally distributed variables, and Spearman correlation analysis for non-normally distributed variables. A p<0.05 was considered statistically significant.

## Results

One hundred and ten subjects took part in the study, divided into 62 obese patients and 48 healthy controls. In the whole cohort the ages ranged from 10 to 18 years. MetS was identified in 45.1% (28/62) of the obese group, but in no members of the control group. Mean ages were 13.5±3.5 years in the control group (group 1, n=48), 13.8±2.9 in the MetS- obese subjects (group 2, n=34), and 14.1±3.3 in the MetS+ obese subjects (group 3, n=28). No statistically significant difference was observed between the groups in terms of age, sex distribution, or pubertal stages (p>0.05).

BMI-SDS was 3.0±0.4 in the obese group and 0.5±0.4 in the control group (p<0.001). Mean fasting blood glucose values were within normal limits and not different between the obese and control groups (controls 81.7±8.7 mg/dL, all obese patients 85.3±9.9 mg/dL; p=0.65). BMI-SDS and WC-SDS were significantly higher in the obese cases than in the control group (p<0.001). Morning fasting insulin and HOMA-IR values were also significantly higher than in the controls (obese and control group fasting insulin 20.6±10.8 vs 8.3±3.1 mIU/mL, respectively, p=0.02; HOMA-IR 4.7±2.7 vs 1.8±0.8, respectively, p=0.01). There was no difference in serum lipid values between the two groups (p>0.05). Although NLR and PLR were higher in obese cases than in the controls, the difference was not statistically significant (p>0.05). SIII values were higher in both obese groups, and particularly in the MetS+ subjects, compared to the control group (p=0.021). Comparison of clinical and laboratory data for the MetS- and MetS+ obese subgroups and the healthy control group are shown in Table 1.

Evaluation of central, inner circle and outer circle macular thickness and volume revealed no significant difference between the sexes or between the two eyes (p>0.05). When the thickness and volume of the whole macular retinal region was evaluated, no statistically significant difference was found between the groups (Tables 2, 3).

Investigation of the relationship between clinical and laboratory variables and MRT and MRV revealed no significant association between mean MRT and MRV and age, pubertal stage, BMI-SDS, WC-SDS, systolic/diastolic blood pressure, NLR, PLR or SIII in the control group. No significant difference was observed between age, pubertal stage, systolic/diastolic blood pressure, triglyceride, HDL cholesterol, or PLR in the MetS+ and MetS- obese groups (p>0.05). MRT and MRV values decreased significantly as BMI-SDS and WC-SDS increased in the MetS- and MetS+ obese groups (p<0.05). In contrast to the other groups, in the MetS+ obese group mean MRT (r=-0.326, p=0.007) and MRV (r=-0.303, p=0.007) values decreased significantly as HOMA-IR values increased. The decrease in MRT (r=-0.544, p=0.046) and MRV (r=-0.651, p=0.031) in the MetS+ obese group was also negatively correlated with NLR. Mean MRT and MRV decreased as SIII increased in all obese subjects (p<0.05). The results of Pearson correlation analysis of MRT and MRV and clinical and laboratory data are shown in Table 4.

## Discussion

Obesity and MetS associated with chronic inflammation can affect most systems in the body (1,2,20). This chronic inflammation also has the potential to produce changes in the retina and macular layer (20). Indeed, previous studies have shown that obesity reduces the thickness of the retinal nerve fiber layer in children and affects choroid tissue (21,22,23). Some studies have shown that obesity leads to changes in the macular layers in children. Although the methods and macular layers investigated differ in all these studies, it may nevertheless be concluded that obesity results in macular variability and damage in the pediatric age group (24,25,26). One animal study involving a rodent MetS and impaired glucose tolerance model demonstrated developmental retinal degeneration using microscopy and immunohistochemical methods (27).

To the best of our knowledge, ours is the first study to show macular changes in MetS+ children and the relationship between that variability and metabolic and inflammatory parameters. In the present study, mean MRT and MRV values decreased as BMI-SDS increased in the all obese groups (p<0.05). In addition, negative correlation was found between BMI-SDS and both MRT and MRV values in MetS - obese and MetS + obese groups (Table 4). Negative correlation with BMI-SDS was determined only in the obese group. No correlation with BMI-SDS was observed in the control group. Thus mean MRT and MRV decreased as the proportion of adipose tissue contributing to body composition increased.

Increased adipose tissue in obese cases may cause chronic systemic inflammation and microvascular damage. Vascular endothelial damage, oxidative stress, and chronic inflammation can impair the permeability and supply of microvascular structures which in turn may leads to oxidative stress and hypoxia in tissues. In addition, changes in leptin and adipokine levels, adipose tissue dysfunction, and IR can also develop. The production of inflammatory cytokines and reactive oxygen species increases. Apoptosis and tissue necrosis may then be triggered. Studies have shown that oxidative stress may be an important factor in cell death (1,2,28,29,30). Our results demonstrate that increased adipose tissue appeared to affect MRT and MRV and resulted in thinning of the macula. NLR, PLR and SIII, which can be simply and inexpensively calculated from complete blood count, have been shown to indicate subclinical inflammation in several previous studies (8,31,32,33). Furuncuoglu et al (33) showed that NLR, PLR, and SIII are positively correlated with BMI in adults. Another study of 26,016 adult patients determined positive correlation between NLR and MetS and obesity-related anthropometric data (32). One study of obese children with sleep apnea syndrome, a condition capable of leading to chronic hypoxia, showed that NLR and PLR increased with obesity (34). No previous studies have investigated changes in NLR, PLR, and SIII in children with MetS and ours is the first study to investigate this in children and adolescents. Although these inflammatory markers were higher in all the obese cases in our study group compared to controls, the differences were not statistically significant. However, SIII values were higher in both obese groups, and particularly the MetS+ subjects, than in the control group (p=0.021). Ours is also the first study to investigate macular variability and metabolic parameters and inflammatory markers in children. In contrast to the other groups, in the obese group with MetS mean MRT and MRV were significantly negatively correlated with increasing HOMA-IR values. The decrease in mean MRT and MRV in the MetS+ subjects was also negatively correlated with NLR. Mean MRT and MRV also significantly decreased as SIII increased in all our obese subjects.

### Study Limitations

There are a number of limitations to this study. Plasma levels of inflammatory mediators such as adiponectin, leptin, and interleukin-6 were not measured. However, NLR, PLR and SIII values have recently been shown to reflect chronic subclinical inflammation, were investigated easily and inexpensively. In addition, due to the cross-sectional nature of our study, we were unable to determine whether weight loss and a decrease in adipose tissue would produce any positive change in MRT and MRV, particularly in obese children with MetS. Long-term, prospective observational studies in which weight control is established and inflammation reduced would be needed to show if there was any improvement in markers of macular tissue health with successful management of obesity and MetS.

## Conclusion

In conclusion, this study demonstrated that mean MRT and MRV values decreased as BMI-SDS and WC-SDS increased in MetS+ obese children and adolescents. Mean MRT decreased as HOMA-IR values, a marker of IR, increased in children with MetS. Increased SIII and NLR are associated with macular damage in MetS+ children and may be useful markers of chronic inflammation. Further long-term observational studies with larger participant numbers are now needed to confirm the results of this study.

## Figures and Tables

**Table 1:**
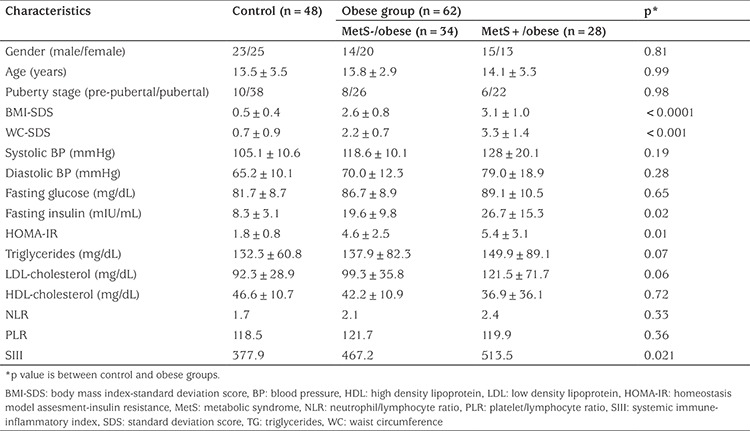
Clinical and laboratory characteristics of the study groups

**Table 2:**
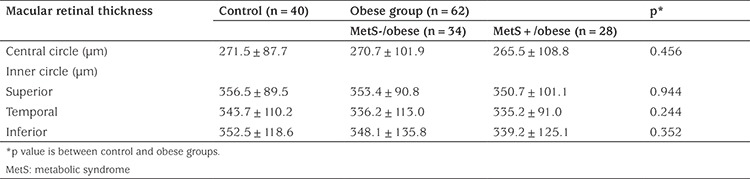
Macular retinal thickness in control and obese children with and without metabolic syndrome

**Table 3:**
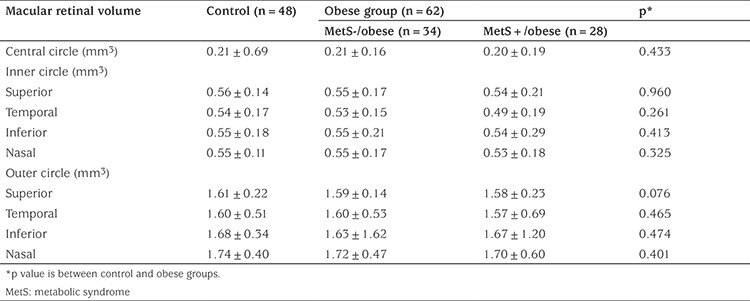
Macular retinal volume in control and obese children with and without metabolic syndrome

**Table 4:**
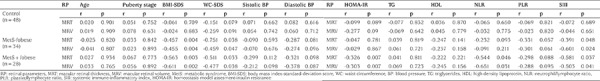
Correlation analysis of macular retinal thickness and macular retinal volume with clinical and laboratory parameters in the study groups
